# Supplementary material for: Image-Guided Brachytherapy for Salvage Reirradiation: A Systematic Review
Source: Cancers (Basel). 2021 Mar 11;13(6):1226. doi: 10.3390/cancers13061226 (PMC7999189; doi:10.3390/cancers13061226)
Supplement: Supplementary file 1 [file cancers-13-01226-s001.zip › Supp Table S1.docx]

# *Supplementary Table S1*. Reirradiation 3D-IGBT studies: prior RT treatment specification and implication of EBRT in reirradiation course.

| **Study** | **Gyn ReRT (n)** | **Prior EBRT only** | | **Prior BT only** | | **Prior EBRT + BT** | | **Prior RT**  **Total Doses (EQD2)**  **Median (range)** | **EBRT allowed before**  **re-RT 3D-IGBT?** | **EBRT doses, if allowed**  **Median (range)** |
| --- | --- | --- | --- | --- | --- | --- | --- | --- | --- | --- |
|  |  | **No of pts** | **Doses (EQD2)**  **Median (range)** | **No of pts** | **Doses (EQD2)**  **Median (range)** | **No of pts** | **Doses (EQD2)**  **Median (range)** |  |  |  |
| Raziee,2020 [32] | 26 | 10 (38.5%) | NR | 0 | 0 | 16 (61.5%) | NR | 63Gy (32.5-89.2) | No | 0 |
| Ling*,* 2019 [44] | 22 | 5 (22.7%) | 45Gy (30.6-50.4) | 12 (54.5%) | 21Gy (10-30)  in 3 Fr ( 2-6) | 5 (22.7%) | NR | NR | Yes  11 pts (50%) | 30.6Gy in 1.8Gy/ Fr in vagina & para-vagina;  Sequential 50.4Gy 1.8Gy/Fr boost in lymph nodes; |
| Umezawa*,* 2018  [41] | 18 | 9 (50%) | 50.4Gy (50-60) | 1 (5.6%) | 30Gy in 5Fr | 8 (44.4%) | EBRT: 50Gy (48.6-50)  BT: 20Gy (12-30) in 4 Fr (3-5) | NR | Yes  5 pts (27.8%) | 40Gy (10-50), 2Gy/Fr |
| Martinez-Monge, 2014  [31] | 15 | 6 (40%) | 54.9Gy (45-60) | 0 | 0 | 9 (60%) | EBRT: 45Gy (45-50.4)  BT: 22Gy (8.5-30) in  3 Fr (range, 1-5) | NR | No | 0 |
| Feddock*,* 2017  [45] | 42 | 11 (26.2%) | 58.4Gy (53-91) | 1 (2.4%) | 35.7Gy | 30 (71.4%) | Median EQD2 dose  88.8Gy (range, 59.5-98.2) | NR | Yes  10 pts (23.8%) | Median dose  22.5Gy (range, 20-45) in 10 Fr (range, 10-25) |
| Liu, 2016 [36] | 16 | 16 (100%) | 45Gy in 1.8Gy/Fr | 0 | 0 | 0 | 0 | 45Gy in 1.8Gy/Fr | No | 0 |
| Mahantshetty*,* 2014 [40] | 30 | 5 (16.7%) | 50Gy (36-63) | 0 |  | 25 (83.3%) | EBRT 50Gy  BT 27Gy (21-49) | NR | No | 0 |
| Zolciak-Siwinska, 2014  [43] | 20 | 5 (25%) | 56Gy (50-64) | 2 (10%) | NR | 13 (65%) | 89.65Gy (49-123) | NR | Yes,  3 pts (15%) | 38 to 52.2Gy, 1.8Gy/Fr (tumor +/- pelvic lymph nodes) |
| Mabuchi, 2014  [33] | 52 | NR | NR | NR | NR | NR | NR | NR | No | 0 |
| Kamran*,* 2017  [39] | 24 | 4 (16.7%) | 44.3Gy ( 23-44.3) | 8 (33.3%) | 29.8Gy | 12 (50%) | 58.5Gy | NR | Yes,  16 pts (66.7%) | NR |
| Murakami*,* 2016  [37] | 8 | NR | NR | NR | NR | NR | NR | NR | NR | NR |
| Amsbaugh, 2015  [34] | 18 | 16 (88.9%) | 45Gy | 2 (11.1%) | NR | 0 | 0 | NR | Yes,  10 pts (55.6%) | NR |
| Yoshida*,* 2015  [42] | 21 | NR | NR | NR | NR | NR | NR | NR | No | 0 |
| Aridgides, 2016  [38] | 6 | 6 (100%) | 50.4Gy (45-64.8) | 0 | 0 | 0 | 0 | 50.4Gy (45-64.8) | Yes,  1 pts (16.7%) | 30Gy, 2Gy/Fr |
| Huang, 2016  [35] | 16 | 5 (31%) | 45Gy | 3 (19%) | 15 to 21Gy in 3 Fr | 8 (50%) | EBRT: 45Gy  BT: 15 to 21Gy in 3 Fr | NR | No | 0 |

Legend: EBRT=external beam radiation therapy; RT=radiation therapy; 3D-IGBT=3D image guided brachytherapy; BT=brachytherapy; EQD2= 2-Gy equivalent doses per fractions; NR=not reported.
