# Supplementary material for: Image-Guided Brachytherapy for Salvage Reirradiation: A Systematic Review
Source: Cancers (Basel). 2021 Mar 11;13(6):1226. doi: 10.3390/cancers13061226 (PMC7999189; doi:10.3390/cancers13061226)
Supplement: Supplementary file 1 [file cancers-13-01226-s001.zip › Supp Text S1_PICO Worksheet and Search Strategy Protocol.docx]

# Supplementary Text S1. PICO worksheet and search strategy protocol

**PICO Worksheet and Search Strategy Protocol: Image-guided brachytherapy for salvage reirradiation: A systematic review.**

**1. Define your question using PICO by identifying: Patient/Problem, Intervention, Comparison group and Outcome:**

Patient/Problem: Local recurrences from gynecologic cancers occurring in previously irradiated field, in non-operable or in patients refusing surgery.

Intervention: 3D image guided brachytherapy

Comparison: None

Outcome: toxicity and efficacy; brachytherapy techniques & dosimetric parameters

Write out your question: To review the recent literature on studies reporting local recurrences from gynecologic cancers, occurring in previously irradiated field, treated by 3D-IGBT, and describe the outcome and toxicities associated to such treatment, as well as brachytherapy techniques used by the authors.

**2. Type of question/problem:**

Therapy

**3. Type of studies/publications to include in the search:**

- Randomized controlled trial

- Research studies or articles

- Research report or other grey literature

**4. List main topics and alternate terms from your PICO question that can be used for your search:**

- Local recurrence from pelvic malignancies/cancer/neoplasm

- Pelvic recurrence

- Vaginal recurrence

- Vulvar recurrence

- Pelvic malignancies

- Gynecological neoplasm

- Gynecologic malignancies

- Gynecologic cancer

- Reirradiation

- Brachytherapy, 3D image guided brachytherapy

**5. Write out your search strategy:**

We will search the literature to identify published articles that reported local recurrences from gynecologic cancers, occurring in previously irradiated field, treated by 3D-IGBT, from 2000 to 2020.

We will use the following search terms and boolean operators: (“pelvic recurrence” OR “vaginal recurrence” OR “vulvar recurrence” OR “recurrent pelvic malignancies”) AND (“gynecologic neoplasm” OR “gynecologic malignancies” OR “gynecologic cancer”) AND “brachytherapy” AND “reirradiation”.

The duplicated will be removed, and the titles of articles will be then evaluated. Abstracts found to be relevant to the topic of interest will be shortlisted. Then full-length papers of the shortlisted articles will be assessed for the eligibility criteria. The articles that fulfilled the criteria will be finally selected for the systematic review.

The inclusion criteria will be as follows: 1) studies reporting patients with local recurrence from gynecologic cancer treated with 3D-IGBT (High Dose Rate (HDR), Pulse Dose Rate (PDR) or Low Dose Rate (LDR) BT), in a reirradiation setting; 2) all type of studies (including randomized controlled trials, prospective, retrospective and case series) comprising more than 5 patients; 3) studies written in English; 4) studies published from January 2000 to December 2020.

Exclusion criteria will be: 1) Studies that did not specifically report brachytherapy reirradiation outcome/toxicity/dosimetric data; 2) Studies with exclusive 2-dimensional treatment planning brachytherapy.

Shortlisted abstract found relevant to the topic of interest and full-length articles fulfilling inclusion criteria will be reviewed independently by two authors (SB and SE) and any disagreement will be resolved by consensus with a third author (CC).

Reasons for excluding studies will be documented.

Following data will be extracted: First author, date of publication, number of patients included, number of patients treated with reirradiation brachytherapy, type of primary disease, type of recurrence, previous treatment modality, treatment free interval, recurrent tumor volume, brachytherapy technique, dosimetric parameters, median follow-up, outcome and toxicities.

**6. List any limits that may apply to your search:**

Gender: female

Age: any

Year(s) of publication: from 2000 to 2020

Language(s): English

**7. List the databases you will search:**

PubMed

Google Scholar
